# Genomic Evidence That Governmentally Produced *Cannabis sativa* Poorly Represents Genetic Variation Available in State Markets

**DOI:** 10.3389/fpls.2021.668315

**Published:** 2021-09-14

**Authors:** Daniela Vergara, Ezra L. Huscher, Kyle G. Keepers, Rahul Pisupati, Anna L. Schwabe, Mitchell E. McGlaughlin, Nolan C. Kane

**Affiliations:** ^1^Kane Laboratory, Department of Ecology and Evolutionary Biology, University of Colorado Boulder, Boulder, CO, United States; ^2^Austrian Academy of Sciences, Vienna Biocenter, Gregor Mendel Institute, Vienna, Austria; ^3^School of Biological Sciences, University of Northern Colorado, Greeley, CO, United States

**Keywords:** cannabinoids, copy number variation, genome diversity, HEMP, repetitive genomic content, marijuana, NIDA, THC

## Abstract

The National Institute on Drug Abuse (NIDA) is the sole producer of *Cannabis* for research purposes in the United States, including medical investigation. Previous research established that cannabinoid profiles in the NIDA varieties lacked diversity and potency relative to the *Cannabis* produced commercially. Additionally, microsatellite marker analyses have established that the NIDA varieties are genetically divergent form varieties produced in the private legal market. Here, we analyzed the genomes of multiple *Cannabis* varieties from diverse lineages including two produced by NIDA, and we provide further support that NIDA’s varieties differ from widely available medical, recreational, or industrial *Cannabis*. Furthermore, our results suggest that NIDA’s varieties lack diversity in the single-copy portion of the genome, the maternally inherited genomes, the cannabinoid genes, and in the repetitive content of the genome. Therefore, results based on NIDA’s varieties are not generalizable regarding the effects of *Cannabis* after consumption. For medical research to be relevant, material that is more widely used would have to be studied. Clearly, having research to date dominated by a single, non-representative source of *Cannabis* has hindered scientific investigation.

## Introduction

Public perception of recreational and medicinal *Cannabis sativa* L. (marijuana, hemp) use has shifted, with *Cannabis* derived products quickly becoming a multibillion-dollar legal industry. However, the National Institute on Drug Abuse (NIDA), a United States governmental agency, continues to be the sole producer of *Cannabis* for research. Additionally, high-tetrahydrocannabinol (THC) producing *Cannabis* continues to be classified as a Schedule I drug, along with heroin, LSD, and ecstasy, according to the DEA (DEA, 2020). This Schedule I classification restricts the acquisition of *Cannabis* from the private markets, making NIDA the only federally legal source for research. In addition to this limitation, research on *Cannabis* requires a multitude of permits and supervision (Nutt et al., 2013; Hutchison et al., 2019). However, the medical and recreational *Cannabis* industry in North America are predicted to grow to 7.7 and 14.9 billion dollars, respectively, by late 2021 (Hutchison et al., 2019).

*Cannabis sativa* (marijuana, hemp) is an angiosperm member of the family Cannabaceae (Bell et al., 2010). It appears to be one of the oldest domesticated plants, utilized by numerous ancient cultures, including Egyptians, Chinese, Greeks, and Romans (Li, 1973, 1974; Russo, 2007). This versatile plant has many known uses, including fiber for paper, rope and clothing, oil for cooking and consumption, and numerous medicinal applications. The plant produces secondary metabolites known as cannabinoids that interact with the human body in physiological (Russo, 2011; Swift et al., 2013; Volkow et al., 2014) and psychoactive (Russo and John, 2003; ElSohly and Desmond, 2005) ways. The cannabinoids compounds are manufactured in the trichomes, which are abundant on the female flowers (Sirikantaramas et al., 2005). The remarkable properties of cannabinoids are partly responsible for driving the growth of the thriving *Cannabis* industry. Two of the main cannabinoids— Δ-9-tetrahydrocannabinolic acid (THCA) and cannabidiolic acid (CBDA)—when heated are converted to the neutral forms Δ-9 THC and cannabidiol (CBD), respectively (Russo, 2011). Two well-characterized enzymes, Δ-9-tetrahydrocannabinolic acid synthase (THCAS) and cannabidiolic acid synthase (CBDAS), are responsible for the production of these cannabinoids in the plant.

Despite the regulatory hurdles and the limited scope of contributions from the United States government, *Cannabis* research is growing at a rapid pace (Vergara et al., 2016; Kovalchuk et al., 2020) and United States scientists have made significant advances in *Cannabis* research from multiple disciplines. Researchers in the United States have produced one of the most complete publicly available *Cannabis* genome assemblies to date, along with the locations of the cannabinoid family of genes in the genome (Grassa et al., 2018). However, oversight is needed to assure the quality and consistency of *Cannabis* testing across laboratories (Jikomes and Zoorob, 2018). Regulation and supervision will allow for a deeper understanding of all the compounds produced by the plant, particularly minor cannabinoids which are not always measured (Vergara et al., 2020) and are produced using multiple genes with complex interactions (Vergara et al., 2019). This is particularly important because medical *Cannabis* use has outpaced its research (Hutchison et al., 2019). Collaborative research between American academic institutions and private companies has shown that the cannabinoid content and genetic profile of *Cannabis* provided by NIDA is not reflective of what consumers have access to from the private markets (Vergara et al., 2017; Schwabe et al., 2019). Therefore, research with these varieties may not reflect the physiological effects of *Cannabis* consumed by the general public.

In 2017, we compared the cannabinoid chemotypes from the *Cannabis* produced in the private market to the chemotypes from the governmentally produced *Cannabis* for NIDA by the University of Mississippi (Vergara et al., 2017). We found that NIDA’s *Cannabis* lacked potency and chemotypic variation and had an excess of cannabinol (CBN), which is a degradation product of THC. The cannabinoid diversity from the governmentally produced *Cannabis* was a fraction (only 27% of the THC) of that from the private markets. A study using microsatellite markers also showed that NIDA’s *Cannabis* was genetically different from commercially available recreational and medical varieties. This study concluded that results from research using flower material supplied by NIDA may not be comparable to consumer experiences with *Cannabis* from legal private markets (Schwabe et al., 2019).

Here, we present results of analysis to further examine the genetic diversity in governmentally produced *Cannabis.* We acquired DNA from two NIDA-produced samples which had been previously analyzed using ten variable microsatellite regions (Schwabe et al., 2019). After sequencing, we compared their overall genomic diversity to that of previously sequenced varieties including hemp- and marijuana-types (Lynch et al., 2016; Vergara et al., 2019). We report here the genomic characteristics of the two NIDA samples, including overall genetic variation, as well as genetic variation within the cannabinoid family of genes, the maternally inherited organellar genomes (mitochondrial and chloroplast), and the repetitive genomic content. We compare this diversity to the publicly available genomes from other *Cannabis* lineages within the species, to characterize the relationships with other well-studied lineages.

## Materials and Methods

### NIDA’s Samples

Bulk *Cannabis* supplied for research purposes is referred to as “research grade marijuana” by NIDA and is characterized by the level of THC and CBD (NIDA, 2016). They offer 12 different categories of *Cannabis* for research that vary in the levels of THC (low < 1%, medium 1–5%, high 5–10%, and very high > 10%) and CBD (low < 1%, medium 1–5%, high 5–10%, and very high > 10%)”. The high THC NIDA sample (Supplementary Table 1) has an RTI log number 13494–22, reference number SAF 027355 and the high THC/CBD has an RTI log number 13784-1114-18-6, reference number SAF 027355. DNA from both samples was extracted by Schwabe et al. (2019) and provided to the University of Colorado Boulder. These two samples were sequenced using standard Illumina multiplexed library preparation protocols as described in Lynch et al. (2016) which yielded to an approximate coverage of 17–20x (Supplementary Table 1).

### Genome Assembly, Whole Genome Libraries, and Nuclear Genome Exploration

We aligned sequences from 73 different *Cannabis* plants to the previously developed CBDRx assembly Cs10 (Grassa et al., 2018). These genomes were sequenced using the Illumina platform by different groups (Supplementary Table 1) and are, or will be, publicly available on GenBank. For detailed information on sequencing and the library preparation of the 57 genomes sequenced by our group at the University of Colorado Boulder please refer to Lynch et al., 2016. The remaining 16 genomes were sequenced and provided by different groups (Supplementary Table 1), however, most of these genomes have been previously used in other studies (Lynch et al., 2016; Vergara et al., 2019).

We aligned the 73 libraries to the CBDRx assembly using Burrows-Wheeler alignment (ver. 0.7.10-r789; Li and Durbin, 2009), then calculated the depth of coverage using SAMtools (ver. 1.3.1-36-g613501f; Li et al., 2009) as described in Vergara et al. (2019). We used GATK (ver. 3.0) to call single nucleotide polymorphisms (SNPs). We filtered for SNPs lying in the single-copy portion of the genome (Lynch et al., 2016) which resulted in 7,738,766 high-quality SNPs. The single-copy portion of the genome does not include repetitive sequences such as transposable elements or microsatellites. Subsequently, we were then able to estimate the expected coverage at single-copy sites as in Vergara et al. (2019). We performed a STRUCTURE analysis (ver. 2.3.4; Pritchard et al., 2000) with *K* = 3 in accordance with previous research (Sawler et al., 2015; Lynch et al., 2016). With these STRUCTURE results, we then classified the different varieties into four different groupings: Broad-leaf marijuana-type (BLMT), Narrow-leaf marijuana-type (NLMT), Hemp, and Hybrid (Supplementary Table 2). Hybrid individuals had less than 60% population assignment probability to a particular group. We found 12 individuals in the BLMT group, 16 in the Hemp group, 14 in the Hybrid group, and 31 in the NLMT group. We then used SplitsTree (ver. SplitsTree4; Huson, 1998) to visualize relationships among the 73 individuals, VCFtools (ver. 4.0; Danecek et al., 2011) to calculate genome-wide heterozygosity as measures of overall variation, and PLINK (ver. 1.07; Purcell et al., 2007) for a principal component analysis (PCA).

### Cannabinoid Gene Pathway Exploration

Using BLAST, we found 12 hits for putative CBDA/THCA synthase genes in the CBDRx assembly (Supplementary Table 3) with more than 80% identity and an alignment length of greater than 1,000 bp. For this BLAST analysis, we used the CBCA synthase (Page and Stout, 2017), the THCA synthase with accession number KP970852.1, and the CBDA synthase with accession number AB292682.1.

We estimated the gene copy-number (CN) for the cannabinoid genes (Vergara et al., 2019) and calculated summary statistics of the CN for each of the 12 genes by variety (Supplementary Table 1). Differences in the estimated gene CN between the cultivars for each of the 12 cannabinoid synthases gene family were determined using one-way ANOVAs on the CN of each gene as a function of the lineages (BLMT, Hemp, Hybrid, and NLMT), with a later *post hoc* analysis to establish one-to-one group differences using the R statistical platform (R Core Team, 2013).

We used BLAST to search for the two enzymes upstream in the cannabinoid pathway using the methodology from Vergara et al. (2019). We found 1 hit to olivetolate geranyltransferase enzyme, and two hits to olivetolic acid synthase (Supplementary Table 1).

### Maternally Inherited Genomes

We used the publicly available chloroplast (Vergara et al., 2015) and mitochondrial (White et al., 2016) genome assemblies to construct haplotype networks using PopART (ver. 1.7; Leigh and Bryant, 2015) using only variants with a high quality score in the variant call file. The chloroplast and mitochondrial haplotype networks comprised 508 and 1,929 SNPs, respectively.

### Repetitive Genomic Content

We used RepeatExplorer (ver.2; Novák et al., 2010) to determine the repetitive content in 71 of the 73 genomes (Pisupati et al., 2018). We excluded ‘‘Jamaican Lion’’ (NLMT) and ‘‘Feral Nebraska’’ (hemp) genomes due to low-quality reads that led to dubious results. We estimated the repetitive content of the genome and annotating repeat families using custom python scripts^1^.

## Results

### Nuclear Genome Exploration

**FIGURE 1:**
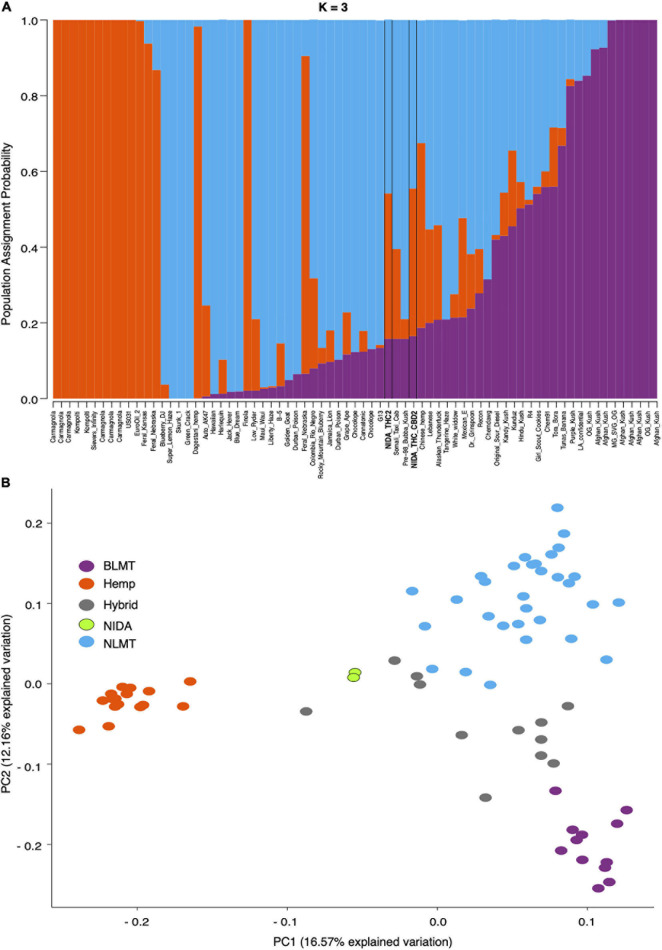
STRUCTURE and Principal Component Analyses. Proportion of each color in the bar indicates the probability of assignment to Hemp (orange), NLMT (blue), or BLMT (purple), groups. Both of NIDA’s strains outlined with black margins are assigned to both NLMT and Hemp groups with less than 60% probability, and therefore we assigned them to the Hybrid group **(A)**. The two NIDA samples in green cluster with each other and away from other varieties **(B)**.

Our analysis of the nuclear genome used 7,738,766 high-quality SNPs from the inferred single-copy portion of the genome. STRUCTURE analysis (Figure 1A) shows the population assignment probabilities for all 73 different varieties including both of NIDA’s varieties. This analysis established that NIDA’s samples cluster with both the hemp and NLMT groupings, with less than 60% in either group, and therefore we categorized them as Hybrid (Supplementary Table 2). The individuals that are part of the Hemp (orange, *n* = 16), NLMT (blue, *n* = 31), or BLMT (purple, *n* = 12) groups had a population assignment probability of more than 60% to that particular group. However, those individuals with a probability of less than 60% to a particular population were assigned to the Hybrid group (gray, *n* = 14), which includes both of NIDAs samples.

**FIGURE 2:**
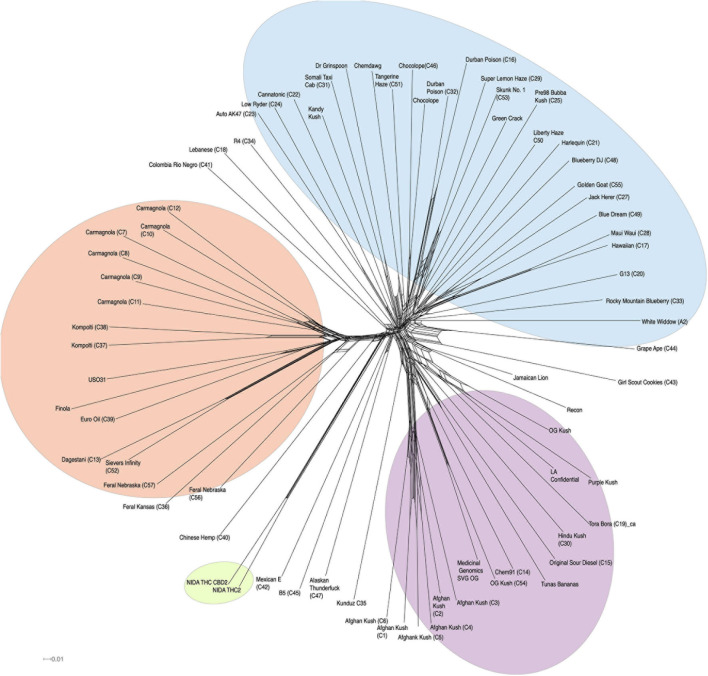
SplitsTree graph. Genetically similar individuals cluster together, such as the NIDA cluster, “Afghan Kush” cluster, and “Carmagnola” cluster. NIDA samples are highlighted in green. Hemp, NLMT, and BLMT shown in orange, blue, and purple, respectively.

In addition to clustering probability results (Figure 1B) from STRUCTURE, we colored the varieties in the PCA (Figure 1B) and SplitsTree (Figure 2) according to their color scheme from the STRUCTURE analysis. The first two principal components in the PCA explain 28.71% of the variation (Figure 1 bottom panel), and the two NIDA varieties cluster together, also seen in the SplitsTree analysis (Figure 2). Both the PCA and SplitsTree indicate high genetic similarity between the NIDA samples and neither of them cluster with any other strains.

**FIGURE 3:**
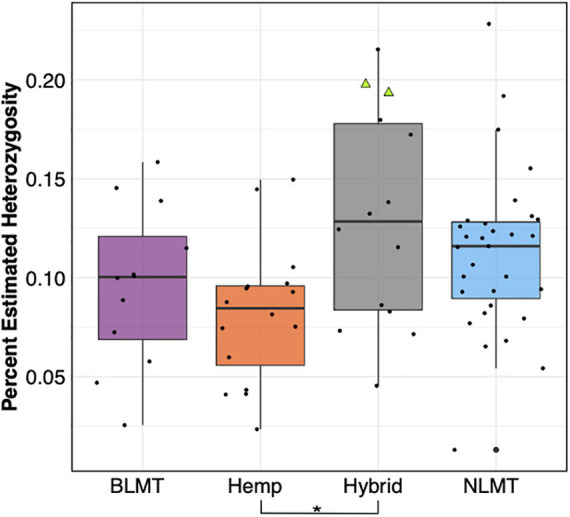
Genome wide heterozygosity. The Hemp lineage differs significantly from the Hybrid grouping with a *P* < 0.03. The two NIDA samples are presented within the Hybrid grouping by two green triangles.

The Hybrid group which contains NIDA’s samples show the widest range of heterozygosity (μ = 0.131, s.d = 0.0545) in the single-copy portion of the genome. However, it is not significantly different from any other group (Figure 3). This wide range of heterozygosity in the hybrid group is expected given that we are grouping individuals that do not belong to one particular genetic group but rather have some assignment probability to two or three genetic groups. Therefore, varieties which are not related to each other, or that belong to more than one group are found in the hybrid category. This may explain why the Hybrid group has the highest mean heterozygosity in this study (Hemp: μ = 0.0817, s.d = 0.0352; BLMT μ = 0.0959, s.d = 0.0405; and NLMT μ = 0.112, s.d = 0.0411).

### Cannabinoid Gene Pathway Exploration

**FIGURE 4:**
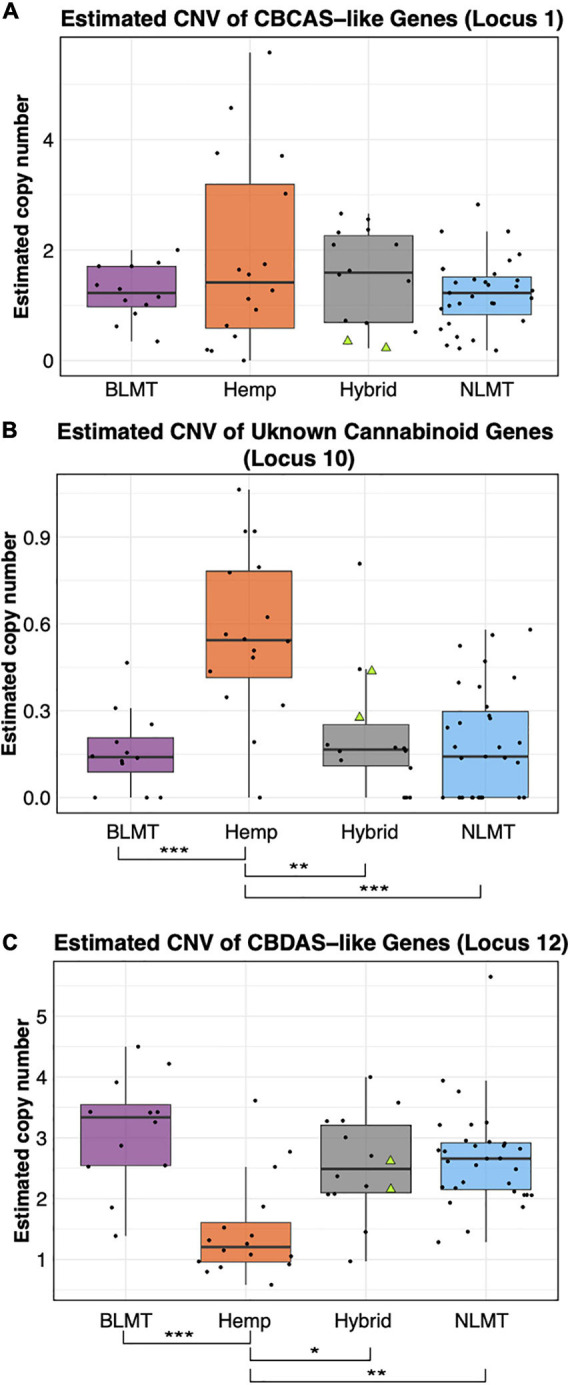
Copy Number Variation in cannabinoid genes. The estimated copy number of the CBCAS-like genes **(A)** is not different between groups despite the Hemp lineage having the widest range. Another unknown cannabinoid locus **(B)** shows significant differences between Hemp and the other groups at the *P* < 0.001 level. The Hemp lineage also differs significantly with a *P* < 0.01 from the other lineages in the estimated copy number of CBDAS-like genes **(C)**. The two NIDA samples are presented within the Hybrid grouping by two green triangles.

Independent of which synthase we used for the BLAST analysis (either THCA, CBDA, or CBCA), the BLAST results delivered the same hits on the CBDRx assembly with different percent identities. Based on percent-identity scores, our BLAST results identified a hit in the CBDRx assembly that appears to code for cannabichromenic acid synthase (CBCAS), and one that possibly codes for CBDAS, but we did not find a hit for THCAS (Supplementary Table 3). After calculating the copy number variation, we found that most groups have one copy of the CBCAS gene (BLMT μ = 1.38, s.d = 1.1; Hemp μ = 1.88, s.d = 2.15; Hybrid μ = 1.56, s.d = 1.33; and NLMT μ = 1.44, s.d = 2.57). Despite the hemp group having the widest range, no group significantly differed from the others (Figure 4A). For the CBCAS genes, the NIDA samples had estimated copy numbers of 0.37 and 0.34. These values are on the lower side of the copy number distribution, with values ranging from 0.016 to 8.75. We include the copy number variation of an unknown cannabinoid, which was the only other locus that had significant differences between groups (Figure 4B).

The copy number variation for the CBDAS gene was higher, ranging from 1 to 3 or more copies (BLMT μ = 3.24, s.d = 1.23; Hemp μ = 1.57, s.d = 1.04; Hybrid μ = 2.59, s.d = 1.17; and NLMT μ = 2.97, s.d = 3.15). The Hemp group on average has a lower copy number of these genes, which is significantly different from every other group (Figure 4C). For the CBDAS genes, the NIDA samples had an estimated copy number of 2.35 and 2.55. These copy number estimates are close to the mean and median values of the whole dataset (μ = 2.64; median = 2.55).

The copy number estimates for the two enzymes upstream in the cannabinoid olivetolate geranyltransferase, and olivetolic acid synthase (Supplementary Table 1) were not significantly different between groups. The approximate copy number for olivetolate geranyltransferase was one gene (BLMT μ = 1.51, s.d = 0.89; Hemp μ = 1.06, s.d = 0.70; Hybrid μ = 1.32, s.d = 0.89; and NLMT μ = 1.89, s.d = 5.53). The approximate copy number for the two copies of olivetolic acid synthase was higher, ranging from 1 to 2 copies (BLMT μ = 0.98, s.d = 0.73; Hemp μ = 0.64, s.d = 0.55; Hybrid μ = 0.57, s.d = 0.46; NLMT μ = 1.24, s.d = 4.41 for the first gene, and BLMT μ = 1.47, s.d = 0.74; Hemp μ = 1.33, s.d = 1.03; Hybrid μ = 1.39, s.d = 0.93; and NLMT μ = 2.00, s.d = 5.79 for the second gene).

### Maternally Inherited Genomes

**FIGURE 5:**
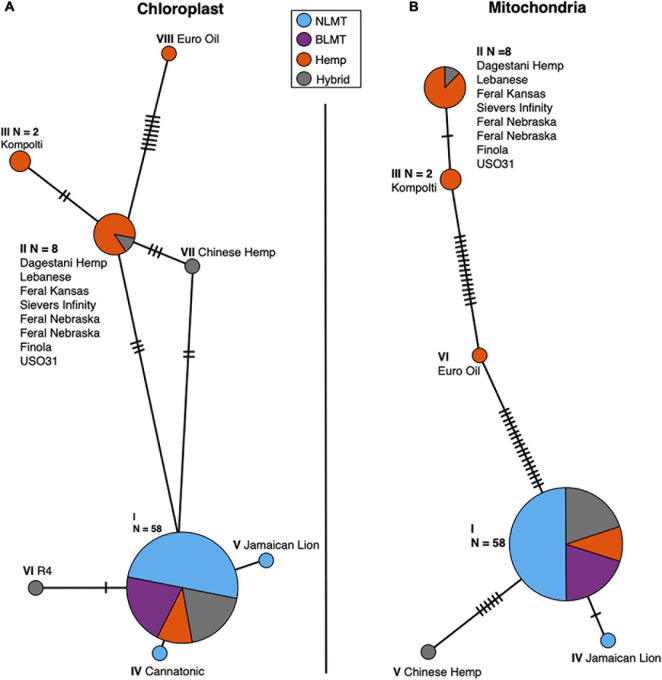
Chloroplast **(A)** and Mitochondrial **(B)** haplotype networks. Both haplotype networks are similar with a common haplotype shared by most individuals (79 and 82% for the chloroplast and mitochondria, respectively) and smaller haplotypes that differ slightly, mostly comprised of Hemp individuals.

We analyzed both the chloroplast (Figure 5A) and mitochondrial (Figure 5B) haplotype networks. The chloroplast haplotype network (Figure 5A) contains eight haplotypes, with a common haplotype (I) that comprises 58 individuals (79%). Most of the individuals in the haplotypes that diverge from the main haplotype (haplotypes II, V, and VI) are hemp types. Both NIDA samples possess the main chloroplast haplotype (I).

The mitochondrial haplotype network contains a common haplotype with 60 individuals (82%), and five additional haplotypes which are mostly comprised of hemp individuals (Figure 5B). As with the chloroplast, both the NIDA samples possess the common haplotype. The haplotype group for each individual for both the chloroplast and mitochondria is given in columns 11 and 12 in Supplementary Table 1.

### Repetitive Genomic Content

**FIGURE 6:**
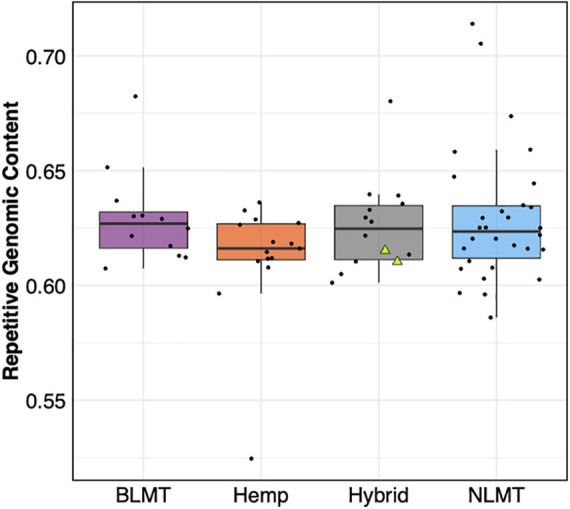
Repetitive Genomic content. The estimated repetitive genomic content by group which does not differ significantly between groups. The two NIDA samples are presented within the hybrid grouping by two green triangles.

We found that the 71 genomes analyzed had similar repetitive content in their genomes (BLMT μ = 62.9%, s.d = 2%; Hemp μ = 61.2%, s.d = 2.6%; Hybrid μ = 62.8%, s.d = 2%; and NLMT μ = 62.9%, s.d = 3%) with few outliers (Figure 6). The NLMT had the most variation in the fraction of genomes containing repetitive content, ranging from 58.6 to 70%. Both NIDA samples (showed as triangles in Figure 6) had 61.1% of their genomes as repetitive content. As shown in Pisupati et al. (2018), the majority of repetitive content in *Cannabis* is composed of Long Terminal Repeats (LTR) elements (Ty1 copia and Ty3 gypsy; Supplementary Figure 1).

## Discussion

In this study, we analyzed the genomes of two *Cannabis* samples produced by the sole legal provider of *Cannabis* for research in the United States, the NIDA. We compared these two samples to the genomes of 71 commercially available varieties, many of which are medicinally or recreationally available on the legal market for sale to the general public. A previous study has shown that *Cannabis* provided by NIDA lacks diversity and cannabinoid potency compared to commercially available *Cannabis* (Vergara et al., 2017), and microsatellite marker analysis also shows that these differences extend to the genetic level (Schwabe et al., 2019). The results of this study concur with previous studies that NIDA-produced *Cannabis* fundamentally differs from *Cannabis* consumed by the public.

Our whole-genome exploration suggests that the samples from NIDA are very similar to each other, and not divergent to all other varieties in our analysis (Figures 1, 2), including the varieties commonly used for recreational and medical purposes (Figure 2). Therefore, the samples from NIDA seem to be distantly related to those that are publicly available for consumption.

Even though the two samples supplied by NIDA have high heterozygosity (Figure 3 and Supplementary Table 1), they are comparable to other varieties from the Hybrid group and from the NLMT group. The high heterozygosity of both samples from NIDA could be due to recent outcrossing, and perhaps a recent hybrid origin. However, because we only sampled two individuals, this may not represent the overall heterozygosity of all varieties produced for NIDA. Still, as already stated, previous research on the chemotypic variation of NIDA’s varieties show their limited cannabinoid diversity (Vergara et al., 2017), supporting the possibility that these two samples are recent hybrids and not bred for their chemotypic profiles including cannabinoids.

The copy number of the cannabinoid genes from the NIDA samples in some cases fall under the median (Figure 4A), above the median (Figure 4B), or near the median (Figure 4C). However, there are some varieties that have up to 13 copies of some genes (Supplementary Table 1), in agreement with previous reports (Vergara et al., 2019). Gene copy number may have implications in cannabinoid production (Vergara et al., 2019), and in gene expression influencing several phenotypes that are also relevant to other plant systems (Stranger et al., 2007; Gaines et al., 2010; Ollivier et al., 2016). Furthermore, since gene expression is correlated with enzymatic activity (Li and Yi, 2012; Xu et al., 2014), it is crucial to understand how gene copy number in the cannabinoid genes is related to enzymatic activity and to cannabinoid production, particularly because varieties and individuals within varieties differ in the number (Vergara et al., 2019) and type of cannabinoid genes (van Velzen and Schranz, 2020). Therefore, future studies once legalization allows for proper *Cannabis* material to be studied at academic research institutions could focus on the expression differences of key cannabinoid genes at the mRNA and proteins levels through transcriptomic and proteomic analyses. However, the observations from this genomic study may be one of the reasons that account for the differences in chemotype between different cannabis varieties, and our study presents evidence that substantiates, at the genomic level, previous findings that the NIDA strains differ chemotypically from *Cannabis* available to the public (Vergara et al., 2017).

Regarding the analysis of the maternally inherited genomes, both NIDA samples have common haplotypes compared to other varieties in the analysis, supporting recent research on the mitochondrial genome diversity in *Cannabis* (Attia et al., 2020). The repetitive content in the samples from NIDA is comparable to that from other varieties (Figure 6), which is mostly still unknown (Supplementary Figure 1). However, NIDA’s samples are in the lower end of the range of repetitive content with 61%. The lack of genetic similarity between NIDA and other strains, as apparent in the genetic clustering illustrated in Figure 1, may explain why the chemotype of NIDA material is different from *Cannabis* from the legal market (Vergara et al., 2017). Other factors contributing to NIDA’s aberrant chemotype could be differences in cultivation, storage, and processing.

One of the caveats of this investigation is that the Hybrid group is not a lineage of truly related individuals, but a grouping of individuals whose population assignment probability is less than 60% to any of the other groups, and hence is somewhat arbitrary. Had we chosen a higher Hybrid assignment probability value, there would be fewer individuals in the NLMT, BLMT, or Hemp groupings and more individuals in the Hybrid group. Had we chosen a lower value, there would be fewer individuals in the Hybrid category and more individuals in the other groupings. However, there are individuals with 100% assignment probability to one group, for example, “Carmagnola” has 100% genetic assignment to the Hemp group, “Afghan Kush” has 100% genetic assignment to the BLMT group, and “Super Lemon Haze” has 100% genetic assignment to the NLMT group. If we had chosen a value of 40% instead of 60%, both the NIDA varieties would have grouped with the NLMT group (see Supplementary Table 2 for assignment probability proportions).

In addition to limiting the research capacity on genetic and chemotypic variation by restricting investigation to only *Cannabis* supplied by NIDA, medical research using this material is also limited. Given that NIDA’s samples do not represent the genomic or phenotypic variation found in *Cannabis* provided by the legal market, consumer experiences may be different from that which is published in the scientific literature. Therefore, medical research is hindered by using varieties that are not representative of what people are consuming, making medical research less predictive. The use of NIDA’s *Cannabis* may be one of the reasons why a recent review found therapeutic support for only three medical conditions (Abrams, 2018), while efficacy as an appetite stimulant, as a relaxant, or to treat epilepsy were not supported despite numerous patient reports (Mattes et al., 1994; Gloss and Vickrey, 2014; Detyniecki and Hirsch, 2016).

Limiting *Cannabis* types available for study creates an obstacle for scientific discovery. It has been proposed that *Cannabis* may be evolving dioecy from monoecious populations (Divashuk et al., 2014; Razumova et al., 2016; Prentout et al., 2019) and cytonuclear interactions, which could be involved in this transition to dioecy, may be also taking place. To understand processes like these, scientists need access to a diverse and growing variety of *Cannabis* plants which are not available through NIDA. Important discoveries in other plant groups, such as transposable elements (McClintock, 1950), genes related to pathogen resistance (Leister et al., 1996), or genes related to yield (Sakamoto and Matsuoka, 2008) would have not been possible had there been similar restrictions on their research.

This limitation also affects the untapped possibilities of using *Cannabis* to treat a multitude of illnesses, with enough anecdotal evidence from consumers to merit rigorous scientific investigation, using strains that are reflective of those used by consumers claiming medicinal and/or therapeutic effects.

*Cannabis* is the most widely consumed illicit substance in both in the United States and worldwide (Gloss, 2014), and therefore it is a matter of public health and safety to provide honest and accurate information. This information is also crucial to policy officials who rely on facts for laws and regulation. In conclusion, scientists must be allowed to use all publicly available forms of *Cannabis* for research purposes to maximize scientific, economic, and medicinal benefit to society.

## Data Availability Statement

The genomic libraries for NIDA1 and NIDA2 are available on NCBI (Accessions SAMN19677471 and SAMN19677472, respectively). The datasets generated and analyzed for this study can be found in the Dryad repository https://doi.org/10.5061/dryad.3n5tb2rgm.

## Author Contributions

DV analyzed the single-copy portion of the genome, made figures, wrote the first draft of the manuscript, and conceived and led the project. EH analyzed the single-copy portion of the genome including STRUCTURE and SplitsTree analyses and wrote the pertinent bioinformatic pipelines. KK wrote bioinformatic pipelines for the single-copy portion analysis and PCA. RP analyzed the repetitive content of the genome. AS and MM acquired DNA samples. NK conceived and directed the project. All authors contributed to manuscript preparation.

## Conflict of Interest

DV is the founder and president of the non-profit organization Agricultural Genomics Foundation, and the sole owner of CGRI, LLC. NK is a board member of the non-profit organization Agricultural Genomics Foundation. The remaining authors declare that the research was conducted in the absence of any commercial or financial relationships that could be construed as a potential conflict of interest.

## Publisher’s Note

All claims expressed in this article are solely those of the authors and do not necessarily represent those of their affiliated organizations, or those of the publisher, the editors and the reviewers. Any product that may be evaluated in this article, or claim that may be made by its manufacturer, is not guaranteed or endorsed by the publisher.

## Footnotes

1
https://github.com/rbpisupati/nf-repeatexplorer.git
