# Supplementary material for: Genomic Evidence That Governmentally Produced Cannabis sativa Poorly Represents Genetic Variation Available in State Markets
Source: Front Plant Sci. 2021 Sep 14;12:668315. doi: 10.3389/fpls.2021.668315 (PMC8476804; doi:10.3389/fpls.2021.668315)
Supplement: Supplementary file 1 [file Data_Sheet_1.docx]

Genomic evidence that governmentally produced *Cannabis sativa* poorly represents genetic variation available in state markets

**Daniela Vergara^1*^, Ezra L. Huscher^1+^, Kyle G. Keepers^1+^, Rahul B. Pisupati^2^, Anna L. Schwabe^3^, Mitchell E. McGlaughlin^3^, and Nolan C. Kane^1*^**

^1^Kane Laboratory, Department of Ecology and Evolutionary Biology, University of Colorado Boulder, Boulder, Colorado, USA

^2^ Gregor Mendel Institute (GMI), Austrian Academy of Sciences, Vienna Biocenter (VBC), Dr. Bohr-Gasse 3, 1030 Vienna, Austria

^3^ University of Northern Colorado, School of Biological Sciences, Greeley, CO 80639, USA.

+Authors that contributed equally

**Supporting Information Figure S1. Repetitive content characterization.** The graph based clustering algorithm Repeat Explorer characterized the percentage of the genome that belong to the different repeat families. Exact numbers in table S4.

**Supporting Information Table S1. Genetic and genomic information.** Cultivar name (column 1), Sample ID (column 2), classification based on Structure (Column 3), NCBI accession number (column 4), provider (column 5), genome calculations (columns 6-10), haplotype groups (columns 11-12), heterozygosity calculations (columns 13-20), PCA (columns 21-40), cannabinoid loci statistics (columns 41-76), olivetolic acid synthase statistics (columns 77-82), olivetolate geranyltransferase statistics (columns 83-85).

**Supporting Information Table S2. Population assignment probability.** Sructure’s population assignment probability. Individuals with an assignment probability of <60% to any group were assigned to the ‘hybrid’ grouping.

**Supporting Information Table S3. Cannabinoid BLAST results.** Cannabinoid BLAST results to the Cs10 assembly with more than 80% identity and an alignment length of greater than 1000bp.

**Supporting Information Table S4. Repeat Families.** Different families based on the clustering algorithm used in Repeat Explorer.
